# Novelty and Novel Objects Increase c-Fos Immunoreactivity in Mossy Cells in the Mouse Dentate Gyrus

**DOI:** 10.1155/2019/1815371

**Published:** 2019-08-27

**Authors:** Hannah L. Bernstein, Yi-Ling Lu, Justin J. Botterill, Helen E. Scharfman

**Affiliations:** ^1^The Nathan S. Kline Institute for Psychiatric Research, Center for Dementia Research, 140 Old Orangeburg Rd., Orangeburg, NY 10962, USA; ^2^Departments of Child and Adolescent Psychiatry, Neuroscience and Physiology, and Psychiatry, and the Neuroscience Institute, New York University Langone Health, 100 First Ave., New York, NY 10016, USA

## Abstract

The dentate gyrus (DG) and its primary cell type, the granule cell (GC), are thought to be critical to many cognitive functions. A major neuronal subtype of the DG is the hilar mossy cell (MC). MCs have been considered to play an important role in cognition, but *in vivo* studies to understand the activity of MCs during cognitive tasks are challenging because the experiments usually involve trauma to the overlying hippocampus or DG, which kills hilar neurons. In addition, restraint typically occurs, and MC activity is reduced by brief restraint stress. Social isolation often occurs and is potentially confounding. Therefore, we used c-fos protein expression to understand when MCs are active *in vivo* in socially housed adult C57BL/6 mice in their home cage. We focused on c-fos protein expression after animals explored novel objects, based on previous work which showed that MCs express c-fos protein readily in response to a novel housing location. Also, MCs are required for the training component of the novel object location task and novelty-encoding during a food-related task. GluR2/3 was used as a marker of MCs. The results showed that MC c-fos protein is greatly increased after exposure to novel objects, especially in ventral DG. We also found that novel objects produced higher c-fos levels than familiar objects. Interestingly, a small subset of neurons that did not express GluR2/3 also increased c-fos protein after novel object exposure. In contrast, GCs appeared relatively insensitive. The results support a growing appreciation of the role of the DG in novelty detection and novel object recognition, where hilar neurons and especially MCs are very sensitive.

## 1. Introduction

The dentate gyrus (DG) is a region within the hippocampus that receives its major input from the entorhinal cortex (EC) via the perforant path (PP) and projects to area CA3 of the hippocampus. Based on this anatomical organization, it has been suggested that the DG contributes to the processing of cortical input before it reaches area CA3. Several possibilities for this role as a “preprocessor” have been suggested, with common views suggesting that the DG “sparsifies” or functions as a “pattern separator” of the diverse input it receives from the EC [1–4]. However, there are additional pathways to both the DG and CA3, making it likely that additional functions are subserved. Many possible functions have been suggested, such as a role of the DG in mood regulation [5–9]. In this study, we focused on the role of the DG in the detection of novel aspects of the environment.

There are multiple lines of evidence to support the role of the DG in novelty detection. Lesion studies suggest that the DG is involved in novelty detection for both large and small environmental changes. Several electrophysiology studies also suggest that synaptic plasticity in the DG GC, the primary cell type, is involved in novelty detection. One study showed that exploration of a novel environment could either enhance or inhibit long-term potentiation (LTP) of PP to GC synapses, depending on the time the LTP protocol was initiated [10]. In another study, placing rats in an environment with novel objects potentiated evoked population spikes at the PP to GC synapse, suggesting enhanced information transmission to the DG in the presence of novelty [11]. Additional studies more recently have shown that the performance of a novel object location task is impaired if adult-born GCs are reduced or impaired [[12, 13] but see [14, 15]], which is interesting because hilar mossy cells (MCs) provide a strong input to the adult-born GCs [16–18].

The potential role of the DG in distinguishing novelty in the environment has received significant attention [10, 11, 19–22]. This role may be similar to pattern separation in that the DG “disambiguates” similar cues, tasks, or experiences [23–25]. Although these studies focus on the GCs, MCs are a significant population of cells in the DG too. However, the importance of MCs to DG functions has been studied much less than the role of GCs. In addition, studying the role of MCs is often complex because they directly excite GCs as well as GABAergic interneurons that inhibit GCs [26]. Notably, the MC axon is the primary afferent input to the proximal third of GC dendrites, so it is likely to be a significant regulator of GCs [27].

Prior studies suggest that MCs have characteristics that make them well-suited to a role in novelty detection. For example, MCs exhibit high levels of spontaneous activity, appear highly sensitive to afferent excitation, and have extensive projections to GCs throughout the septotemporal axis bilaterally [28–30]. These qualities would allow MCs to integrate spatial and sensory PP inputs with additional afferent inputs to inform GCs about environmental changes. As a result, MCs have been suggested to be novelty detectors [26]. It was also recently shown that MCs have flexible place fields compared to GCs [29, 31, 32], suggesting that they might be especially tuned to spatial novelty cues.

The immediate early gene c-fos has been extensively used as a tool to map behaviorally relevant patterns of neural activity [33]. Numerous studies have used c-fos to examine the recruitment of different brain regions, including the hippocampus, in response to multiple types of novel stimuli [34–43]. These studies have led to mixed results, and importantly, none of the studies examining hippocampal c-fos protein expression following the introduction of novel stimuli have specifically focused on the hilus or MCs.

Our laboratory has previously used c-fos to study the effect of environmental manipulations on DG activity. One study performed in the rat used c-fos protein expression to compare MC activation between animals that had been recently moved to a new environment to animals that were more acclimated [44]. A subset of MCs, especially in the ventral DG, was labeled by c-fos even in animals that had acclimation [44], whereas other hippocampal cells had low c-fos protein expression. Because ventral MCs primarily project to dorsal GCs, the results suggested a role of ventral MCs to provide input about environmental novelty to dorsal GCs [44]. A second study looked at the effects of brief restraint stress on MC c-fos in rats, because the DG is sensitive to stress [45, 46] and MCs have stress-sensitive glucocorticoid receptors [47]. It was found that brief restraint caused a transient drop in MC c-fos levels that could be detected as soon as 20 minutes after restraint but then rapidly recovered, suggesting a rapid sensitivity of MCs to environmental change that could aid in novelty detection [48].

Thus, while there is a significant body of evidence suggesting that MCs are activated by novel stimuli, this has not been specifically addressed. Different types of novelty have also not been systematically explored. In addition to detecting novel environments, rodents can discriminate novel objects from those objects that are familiar. To study this phenomenon, researchers have developed the novel object recognition (NOR) task and novel object location (NOL) task (see Methods). Another qualitatively different type of novelty is the novelty of having a new experience—such as performing any of the above tasks for the first time.

In this study, we specifically examined the role of MCs in the detection of novel vs. familiar objects in a paradigm resembling NOR, while limiting environmental and spatial novelty and controlling for new experiences. c-Fos was used as a proxy to measure neural activity in the hilus and GC layer (GCL) across the hippocampal axis. Unlike in previous studies, all experiments were performed in the home cage to eliminate the environmental novelty of a testing arena, and instead isolate object novelty. After putting novel or familiar objects in the home cage of pair-housed mice, we found that MC c-fos was increased from baseline, and that novel objects elicited more hilar c-fos than familiar objects. Animals exposed to objects for the first time (a novel experience) had the highest hilar c-fos, compared to animals who encountered novel objects but had already seen different objects before. Our findings support a role for MCs in the detection of both novel objects and novel experiences.

## 2. Methods

All procedures were approved by the Institutional Animal Care and Use Committee of The Nathan Kline Institute. All chemicals were purchased from Sigma-Aldrich unless otherwise specified.

### 2.1. Animals

Adult male mice between 2 and 5 months old were used. Animals were obtained from Jackson Laboratories (C57BL/6J; stock no. 000664) and bred in-house. Breeders were fed Purina 5008 chow (W.F. Fisher and Son Inc.) and provided 2^″^ × 2^″^ nestlets (W.F. Fisher and Son Inc.) until weaning at 25-30 days of age, and then mice were housed with others of the same sex (2-4/cage) and fed Purina 5001 chow (W.F. Fisher and Son Inc.) until use. At all times, mice were provided food and water ad libitum. They were housed in standard mouse cages with a 12 hr light : dark cycle and relative humidity between 64 and 78%. All behavior was studied during the light phase of the light : dark cycle. Home cages were moved to the laboratory at least 24 hours prior to use to reduce the effects of environmental novelty.

### 2.2. Behavior

#### 2.2.1. General Methods

Group-housed mice were split into pair housing and acclimated to the laboratory for at least 24 hours before testing. The cages were kept in a designated overnight housing area of the laboratory at all times except during object exploration, when the cages were brought to the laboratory bench for 5 minutes. Object exploration was performed between 1:00 pm and 4:00 pm for all animals, and animals were freely allowed to explore the objects. There was no evidence that one animal influenced the exploration of the other; instead, animals remained separated spatially. Objects were cleaned with 70% ethanol and then with water between testing. In pilot studies, we confirmed that a separate group of mice showed no preference for either of the objects used in the present studies (i.e., LEGO objects and plastic swans).

The tasks used in this study were based on NOR and NOL. In the NOR task, mice are exposed to 2 identical objects, then after a delay the original object is presented with a new (novel) object. Rodents and other animals generally prefer to explore the novel object, indicating that they remember the original object [49]. NOR has been shown to be dependent on the hippocampus [49] and appears to involve the DG [50, 51]. The related NOL task involves spatial novelty and requires the animal to identify that an object has been moved instead of replaced [52], and the hippocampus has also been shown to be required for this task [53, 54].

#### 2.2.2. Novel Object Exposure

**Figure 1:**
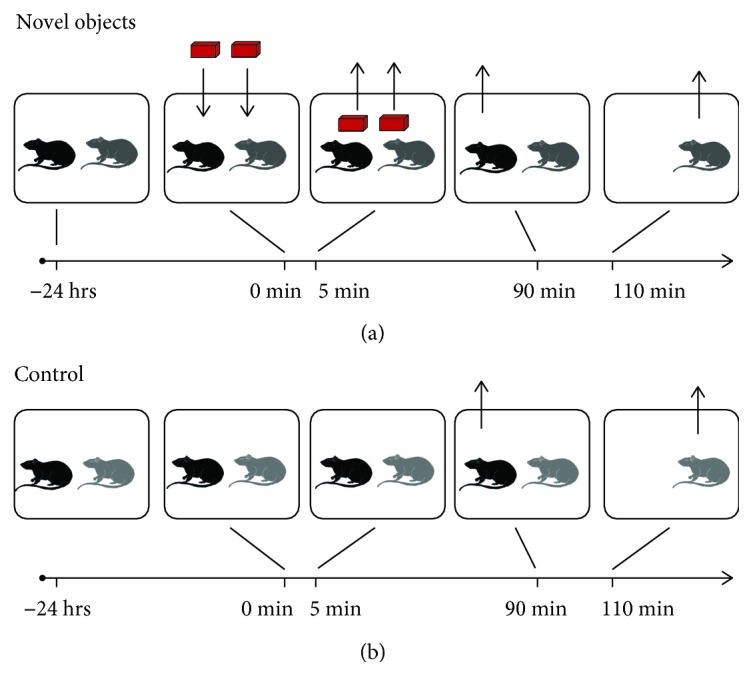
Experimental timeline for the comparison of control and novel object exposure. (a) For animals exposed to novel objects, 2 animals were housed together for over 2 weeks. They were brought to the laboratory and housed there until the next day when they were placed on a lab bench, the cage lid was removed, and 2 identical LEGO objects were placed in the cage center about 6 inches apart. After 5 min, the objects were removed. After 90 min, one animal was perfusion fixed, and 20 min later the remaining animal was perfused. For this series of experiments, mice were not acclimated to objects prior to testing. (b) For control mice, procedures were the same as (a), but objects were not placed in the cage.

For the first series of studies, we evaluated hilar c-fos activity in response to exposure to novel objects compared to baseline c-fos levels, using a design shown in Figure 1. For this series of experiments, mice were not acclimated to objects prior to testing. Cages in the novel object group were placed on a lab benchtop, and 2 identical LEGO objects were put in the cage center, approximately 6 inches apart along the long axis of the cage, for 5 minutes. The cage was then returned to the designated housing area until both animals were perfused, 90 and 110 minutes after the start of object exposure, respectively (Figure 1). These times were selected based on preliminary data from mice that were perfused 30, 90, or between 90 and 110 minutes after the start of object exposure. The c-fos+ cells at 30 minutes were low compared to 90 minutes, whereas c-fos+ cells were similar between 90 and 110 minutes. Cages in the control group were not moved from the housing area until perfusion, with the second animal perfused 20 minutes after the first. Three cohorts were performed at separate times. Note that cohort variation was assessed and it was found that the same direction of the results occurred in all cohorts, although the raw values of c-fos immunoreactive (ir) cells varied from one animal to the next and from one cohort to another.

#### 2.2.3. Exposure to Novel Objects vs. Familiar Objects and Use of Nonhabituated Animals

**Figure 2:**
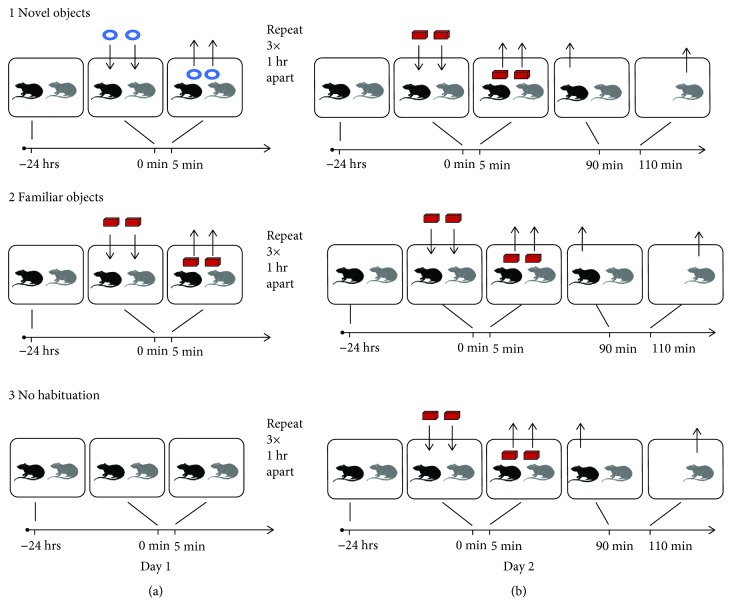
Experimental timeline for the comparison of familiar and novel object conditions. (a,1) For animals exposed to novel objects, 2 animals were housed together for over 2 weeks. They were brought to the laboratory and housed there until the next day, Day 1. On Day 1, the cage lid was removed, and 2 objects were placed in the cage center about 6 inches apart. After 5 min, the objects were removed. This was repeated so that there were 3 exposures for 5 min, one hour apart. (a, 2) For animals exposed to familiar objects, a similar procedure was used, but the objects were different. (a, 3) For animals with no habituation, no objects were presented to the animal on Day 1. (b, 1) For animals exposed to novel objects, procedures on Day 2 were similar to Day 1 but the objects were different. Also, after objects were removed, one animal was perfused 90 min after object exposure began, and the second was perfused at 110 min. (b, 2) For animals exposed to familiar objects, procedures were the same as Day 1. However, object exposure occurred once, and one mouse was perfused 90 min after object exposure and the other was perfused at 110 min. (b, 3) For the no habituation group, novel objects were placed in the cage on Day 2. One mouse was perfused 90 min after object exposure and the other was perfused at 110 min.

The first set of experiments only used novel objects and control conditions, so it did not specifically address the potential role of familiar objects on hilar c-fos activation. Therefore, in the second series of experiments, we compared hilar activation between animals exposed to familiar objects, animals exposed to novel objects, and animals exposed to objects for the first time (no habituation). To do this, we devised a three-group scheme, still using pair-housed mice and objects placed in the home cage, as shown in Figure 2. Two identical pairs of objects were used: object pair A (plastic swans) and object pair B (the LEGO objects from the previous experiments). Preliminary experiments were conducted to show that animals exposed to the 2 types of objects explored each one a similar length of time within a given test period of 5 min. Therefore, animals did not appear to show an inherent preference for one object vs. the other.

In the first group, animals were habituated to object pair A and exposed to object pair B during the test (the novel object group). In the second group, animals were habituated to object pair B and exposed to object pair B during the test (the familiar object group). For the third group, no habituation was performed, and animals were exposed to object pair B for the first time during the test (the no habituation group). For the novel and familiar object groups, object habituation was done the day before (Day 1) the test day (Day 2; Figure 2). Cages in the novel and familiar groups were brought to the bench and exposed to object pairs A and B, respectively, 3 times for 5 minutes each with one hour intervals between exposures. On the Day 2, all 3 groups were exposed to object pair B for 5 minutes. The LEGO objects were always used as object pair B on the test day to maintain consistency with the previous experiments. For all groups, the first animal was perfused 90 minutes after the start of object exposure and the second animal was perfused 20 minutes later. As in the first part of the study, 3 cohorts were performed, resulting in a total of 6 animals per group.

### 2.3. Anatomy

#### 2.3.1. Perfusion Fixation and Sectioning

Animals were deeply anesthetized with isoflurane (by inhalation in a closed glass jar) followed by urethane (2.5 g/kg) intraperitoneally (i.p.). The abdominal cavity was opened, and the animal was transcardially perfused with 10 ml 0.9% NaCl in distilled H_2_O (dH_2_O) followed by 20 ml 4% paraformaldehyde in 0.1 M phosphate buffer (PB; pH 7.4), using a 25-gauge butterfly needle attached to a peristaltic pump (Minipuls 2, Rainin). Brains were removed and postfixed overnight at 4°C. The brain was hemisected and each hemisphere was cut into 50 *μ*m sections using a vibratome (TPI 1000 series, Rankin Biomedical Corp.). The left hemisphere was cut coronally, and the right was cut horizontally. This scheme allows one to study caudal parts of the DG with an orientation that makes the hilar borders with CA3 clear. The commonly used coronal plane shows the hilus in caudal sections, but it is hard to distinguish the hilus from CA3. Sections were collected into the cryoprotectant (25% glycerol, 30% ethylene glycol, and 45% 0.1 M phosphate buffer) and stored at 4°C.

#### 2.3.2. Immunohistochemistry

Brightfield immunohistochemistry for c-fos was conducted as previously described [44]. Every twelfth coronal and horizontal section was used, such that sections were 600 *μ*m apart. Four sections from the most dorsal part of the coronal plane were added to 4 sections from the most ventral part of the horizontal plane (8 total sections per animal) to provide insight into the septotemporal axis of the hippocampus. We use the term dorsal-ventral axis to refer to the data in the Results. Sections were matched for hippocampal level between animals. Washes and incubations were at room temperature on a rotary shaker unless otherwise stated. Free floating sections were washed in 0.1 M Tris buffer (two times, 5 minutes each) and incubated in 1% hydrogen peroxide in 0.1 M Tris buffer for 2 minutes, to block endogenous peroxidases. Sections were then washed again in 0.1 M Tris buffer (3 times, 5 minutes each). After washes, sections were incubated for 10 minutes, first in Tris A (0.25% Triton X-100 in 0.1 M Tris buffer) and then in Tris B (0.25% Triton X-100 and 0.005% bovine serum albumin in 0.1 M Tris buffer). Sections were blocked in horse serum (1 : 400; Vector Laboratories Inc.) in Tris B for one hour to minimize nonspecific binding and then incubated overnight in the primary antiserum (1 : 10,000; goat polyclonal anti-c-fos, SC-52-g, Santa Cruz Biotechnology) at 4°C on a rotary shaker. The next day, sections were washed for 10 minutes in Tris A and then for 10 minutes in Tris B followed by a two-hour incubation with the secondary biotinylated antibody (1 : 400; horse anti-goat, Vector Laboratories Inc.). Sections were washed again for 10 minutes in Tris A followed by 10 minutes in Tris B, then the avidin-biotin-horseradish peroxidase complex (ABC) method for visualization with immunoperoxidase was used [55]. The ABC solution (VECTASTAIN Elite ABC Kit, Vector Laboratories Inc.) was diluted 1 : 100 in Tris B, and the sections were incubated in the diluted ABC solution for one hour. Following washes (3 times, 5 minutes each) in 0.1 M Tris buffer, immunoreactivity was visualized using 3,3-diaminobenzidine (DAB) with NiCl_2_ intensification. Sections were incubated in a solution containing 0.022% DAB (in 0.1 M Tris), 1 mM NiCl_2_ (in dH_2_0), 0.2% ammonium chloride (in dH_2_O), 0.1% glucose oxidase (in dH_2_O), and 0.8% D(+)-glucose (in dH_2_O) in 0.1 M Tris buffer. The reaction was stopped by washing sections in 0.1 M Tris buffer. DAB reactions were stopped at similar levels of background staining between batches. Sections were then washed in 0.1 M Tris buffer and mounted on subbed slides. Slides were allowed to dry overnight and were then dehydrated in a graded series of ethanol (2 minutes in 70% ethanol, 2 minutes in 95% ethanol, and 2 minutes in 100% ethanol 2 times), cleared in xylene (two times, 4 minutes each), and then coverslipped with Permount (Fisher Chemical). All slides were analyzed using a brightfield microscope (BX51, Olympus), photographed using a digital camera (Retiga 2000R, QImaging), and acquired using Image-Pro (Media Cybernetics Inc.).

#### 2.3.3. Double Labeling

Double immunohistochemistry labeling of c-fos and GluR2/3 was performed similarly to that described previously [44, 48]. Sections were first incubated with c-fos primary and secondary antibodies and visualized using DAB intensification, as described above. Next, sections were washed (3 times, 5 minutes each) in Tris and blocked in goat serum (1 : 400, Vector Laboratories Inc.) in Tris B for one hour, then incubated overnight in GluR2/3 primary antibody (1 : 100; rabbit polyclonal anti-GluR2/3, AB1506, Millipore) at 4°C. The next day, sections were washed and incubated in the corresponding biotinylated secondary antibody (1 : 400, goat anti-rabbit, Vector Laboratories Inc.) for 2 hours. Immunoreactivity was visualized with NovaRED per the manufacturer's instructions (Vector Laboratories Inc.; see also [44, 56]). Sections were mounted on slides, dehydrated, coverslipped, and photographed as above.

#### 2.3.4. Quantification

c-Fos immunoreactive (c-fos ir) or c-fos positive (c-fos+) cells in the hilus and GCL were quantified using ImageJ software (NIH) using 12.5x brightfield images. Images were thresholded manually to create a binary overlay in which c-fos+ nuclei were covered just to their borders and background was mainly excluded. The outline of the hilus and GCL was drawn manually, and automated counts of cells based on size and circularity parameters were performed for both areas. We manually confirmed the accuracy of the threshold technique in every section, which we have previously discussed [57]. Quantification of double labeling for c-fos and GluR2/3 in the hilus was performed manually using 40x images.

### 2.4. Statistics

Data are presented as mean ± standard error of the mean (SEM). Significance was set at *p* < 0.05. Power analysis was conducted using StatMate2 (GraphPad) for alpha = 0.05. Based on pilot data, sample sizes were computed for a power of 80%. Comparisons of 2 groups were made using two-tailed unpaired Student's *t*-test for parametric statistics. When parametric conditions were not met, Mann-Whitney's *U* tests were conducted. For more than 2 groups, data were compared using analysis of variance (ANOVA). Repeated measures ANOVA (RMANOVA) was used when comparing matched sections across the dorsoventral axis between animals. For ANOVAs, Bonferroni's or Tukey-Kramer's post hoc tests with correction for multiple comparisons were conducted to assess statistical differences between groups. Statistical comparisons were conducted using Prism (GraphPad).

## 3. Results

### 3.1. Exposure to Novel Objects in the Home Cage Increases MC c-Fos Relative to Control Conditions

For the first series of experiments, c-fos protein expression was examined in animals exposed to objects in the home cage for the first time. For comparison, a control group used animals that were undisturbed in the home cage (Figure 1). We chose 2 identical LEGO objects that we found that the mice readily explored in pilot studies. Two objects were presented instead of one to mimic the conditions of the NOR task, in which animals are exposed to two identical objects in the first phase of the task.

**Figure 3:**
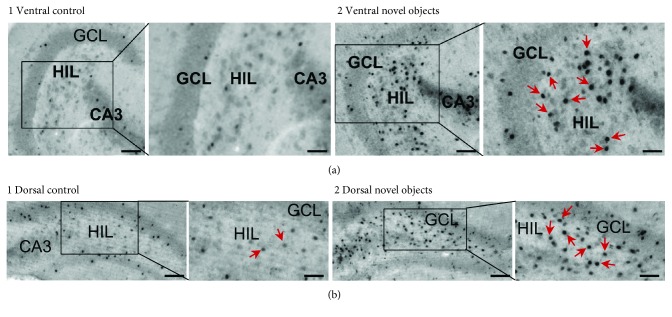
Hilar c-fos+ cells after exposure to novel objects. (a, 1) A section through the ventral hippocampus is shown. There are few c-fos+ cells in the hilus of a control mouse. Calibrations: 75 *μ*m (main image, left) and 50 *μ*m (inset, right). HIL: hilus; GCL: granule cell layer; CA3: area CA3 pyramidal cell layer. (a, 2) In a mouse exposed to novel objects as described in Figure 1, there were many c-fos+ cells in the hilus (arrows). Same calibrations as (a, 1). (b, 1) A dorsal section shows little c-fos+ hilar cells in the control. (b, 2) Numerous hilar c-fos+ cells are present in the dorsal section of a mouse exposed to novel objects (arrows; as described in Figure 1). Calibrations: 75 *μ*m (main image, left) and 60 *μ*m (inset, right).

**Figure 4:**
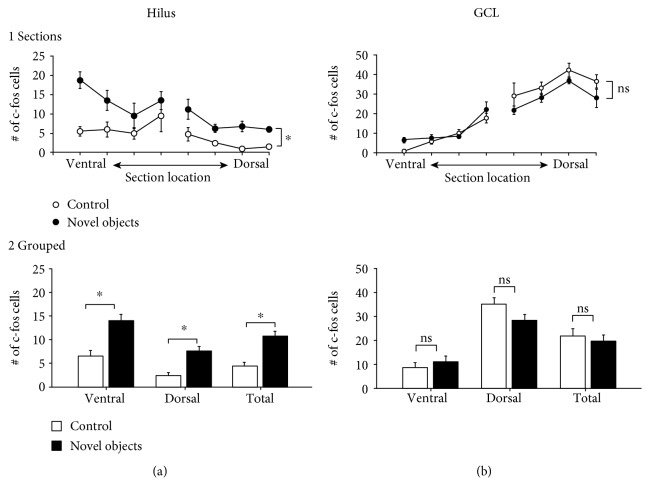
Quantitative differences in hilar c-fos+ cells between control mice and mice exposed to novel objects. (a, 1) The mean number of hilar c-fos+ cells is listed according to their septotemporal location. A RMANOVA showed a significant effect of the behavioral task (control vs. novel object: *F*(1, 70) = 27.90; *p* = 0.0005) and a significant effect of the septotemporal location (relatively ventral or dorsal: *F*(7, 70) = 5.52; *p* < 0.0001) but no interaction of factors (*F*(7, 70) = 1.17; *p* = 0.333). (a, 2) The values for hilar c-fos+ cells in the 4 most ventral and 4 most dorsal sections were pooled and were listed as ventral and dorsal, respectively. The total (ventral+dorsal) is listed as well. A two-way ANOVA showed a significant effect of the behavioral task (control vs. novel object: *F*(1, 20) = 42.35; *p* < 0.0001). There was a significant effect of septotemporal location (*F*(1, 20) = 9.64; *p* = 0.006) with the novel object group showing significantly more ventral than dorsal c-fos protein expression (Tukey's post hoc test, *p* < 0.05) and no interaction of factors (*F*(1, 20) = 2.41; *p* = 0.136). Totals were significantly different (Student's *t*-test; *t* = 5.292; df 10; *p* = 0.0004). (b, 1) The mean number of c-fos+ cells in the GCL is listed according to their septotemporal location (ventral or dorsal). A RMANOVA showed no significant effect of the behavioral task (control vs. novel object: *F*(1, 70) = 1.28; *p* = 0.285) and a significant effect of the septotemporal location (*F*(7, 70) = 46.96; *p* < 0.0001). There was no interaction of factors (*F*(7, 70) = 0.436; *p* = 0.875). (b, 2) The values for GCL c-fos+ cells in the 4 most ventral and 4 most dorsal sections are pooled and are shown; average total values (ventral+dorsal) are also presented. A two-way ANOVA showed no significant effect of the behavioral task (*F*(1, 20) = 1.37; *p* = 0.256). There were more dorsal than ventral c-fos+ GCs (*F*(1,20) = 69.07; *p* < 0.0001; Tukey's post hoc test, *p* < 0.05) and no interaction of factors (*F*(1, 20) = 1.145; *p* = 0.294). Totals were not significantly different (Student's *t*-test; *t* = 1.87; df 10; *p* = 0.091).

Using these conditions, we saw increased numbers of c-fos ir cells in the hilus of mice exposed to novel objects, and this occurred in both ventral and dorsal sections compared to control mice not exposed to novel objects (Figures 3 and 4). By looking at sections across the dorsoventral axis of the hilus, we observed that there were more c-fos ir cells in the ventral hilus than the dorsal hilus both at baseline and in animals exposed to objects (Figure 4(a) 1). In addition, hilar c-fos cell numbers were increased by novel object exposure in every section we examined across the septotemporal axis (Figure 4(a) 1). By pooling ventral sections (sections 1-4) and dorsal sections (sections 5-8), we confirmed that hilar c-fos was statistically higher in object-exposed animals in both the ventral and dorsal hilus (Figure 4(a) 2). Interestingly, this increase in c-fos protein was limited to the hilus, as there was not a significant difference in GCL c-fos ir between groups in the ventral or dorsal DG (Figure 4(b)). These data suggest that hilar neurons are more sensitive to object novelty than cells in the GCL. Interestingly, GCL c-fos was higher dorsally in both groups (Figure 4), which was opposite to the trend observed in the hilus, and is addressed further below when the results of the second series of experiments are presented.

**Figure 5:**
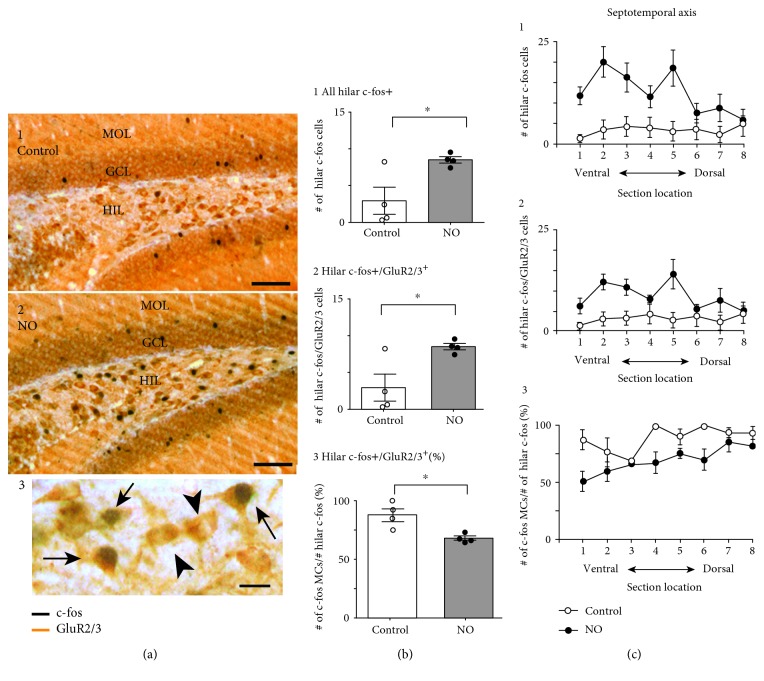
The majority of hilar c-fos+ cells were double labeled for a marker of glutamatergic neurons, GluR2/3, suggesting that they were MCs. (a, 1) A dorsal section from a control mouse double labeled for c-fos (black) and GluR2/3 (orange). Few double-labeled cells are present in the hilus. Calibration: 100 *μ*m. MOL=molecular layer. (a, 2) A dorsal section of a mouse exposed to novel objects shows more double-labeled hilar cells. Calibration: 100 *μ*m. (a, 3) Inset: at higher gain. Double-labeled cells (arrows) and cells only expressing GluR2/3 (arrowhead) are shown. Calibration: 25 *μ*m. (b, 1) The mean value for hilar c-fos+ cells is listed for 4 controls and 4 mice exposed to novel objects (NO). The differences were significant (*t*-test: *t* = 2.37, df 6; *p* = 0.025). (b, 2) The mean value for hilar c-fos+/GluR2/3+ double-labeled cells is shown for the same mice as (b, 1). Differences were significant (*t*-test: *t* = 3.96, df 6; *p* = 0.007). (b, 3) The mean value of hilar double-labeled cells (presumably MCs) as a fraction of all hilar c-fos+ cells is expressed as a percent. Differences were significant (*t*-test: *t* = 3.59, df 6; *p* = 0.011). (c, 1) Values for c-fos+ hilar cells are plotted along the septotemporal axis for 8 sections selected at intervals throughout the axis. These data suggest that the differences in groups were mainly ventral, which was also observed in (c, 2) and (c, 3). Some control sections showed no c-fos+ cells in the hilus, so the most ventral sections and the most dorsal sections were pooled. A two-way ANOVA showed a significant effect of condition (control vs. novel object: *F*(1, 12) = 17.19; *p* = 0.001) and no effect of ventral vs. dorsal position (*F*(1, 12) = 3.78; *p* = 0.076). There was a trend towards an interaction between condition and position (*F*(1, 12) = 4.51; *p* = 0.055) which appeared to underlie a significant difference in post hoc tests comparing ventral location. Thus, Tukey's post hoc test showed a significant difference in the ventral but not dorsal locations.

To identify if the increase in hilar c-fos protein reflected expression in MCs, we performed double labeling for c-fos and GluR2/3. GluR2/3 is a marker of glutamatergic neurons and therefore MCs; other hilar cells are mainly GABAergic [27, 58–60]. Double labeling was done in 4 animals per group, using 8 sections per animal adjacent to those used for labeling using the c-fos antibody alone. Double-stained hilar cells were well labeled with nuclear c-fos in black and cytoplasmic GluR2/3 in orange (Figure 5(a)). Those c-fos ir nuclei that were present in the GCL were double labeled also, and we refer to the c-fos-labeled cells in the GCL below as GCs.

Quantified results showed that the total number of hilar c-fos ir cells was significantly higher in novel object-exposed animals than in controls (Figure 5(b)). In addition, the fraction of all hilar c-fos ir cells that were GluR2/3+ double stained, presumably representing MCs, were also increased after novel object exposure compared to controls (Figure 5(b) 2). However, the fraction of all active hilar cells that were presumable MCs declined in novel object-exposed animals (Figure 5(b) 3). These data suggest that in control conditions, the majority of cells that are c-fos+ are MCs, and while MCs are specifically recruited during novel object exposure, GluR2/3-negative cells of the hilus (possible GABAergic neurons) appear to be activated by novel objects.

### 3.2. Novel Objects Increase MC c-Fos Protein Relative to Familiar Objects, but GC c-Fos Protein Is Relatively Unaffected

The previous experiments demonstrated that hilar neurons, including MCs, are activated by exposure to novel objects in the home cage. To test the hypothesis that the exposure to novel objects was selective, we compared hilar c-fos protein expression between animals exposed to novel or familiar objects. A third group included animals exposed to objects for the first time (“no habituation” group). All mice were pair housed with objects placed in the home cage (Figure 2). Two sets of objects were used. The first set was plastic swans and the second set was LEGO objects described above. These objects were chosen because mice appeared to explore these 2 types of objects equally in preliminary experiments, not displaying an inherent preference.

**Figure 6:**
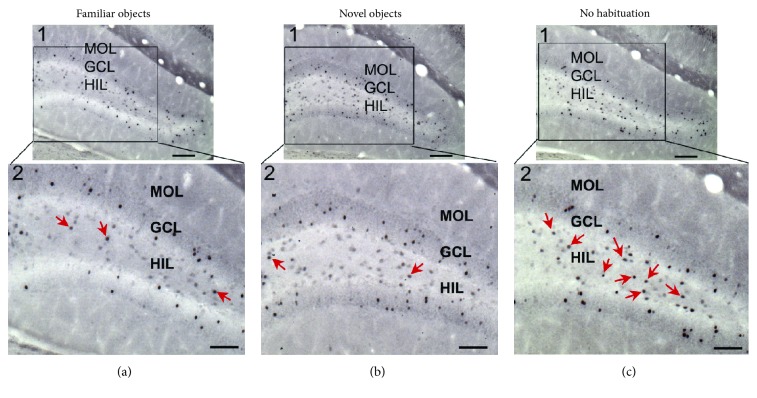
Hilar c-fos labeling is weak in mice exposed to familiar objects compared to novel objects and very strong when mice had not been previously exposed to objects. Three sections are shown with insets showing more detail. The sections are from comparable dorsoventral levels. Mice were either exposed to familiar objects (a) or novel objects (b) or had not been exposed previously to objects (c). Arrows point to c-fos ir hilar cells. Calibrations: 100 *μ*m. MOL=molecular layer.

**Figure 7:**
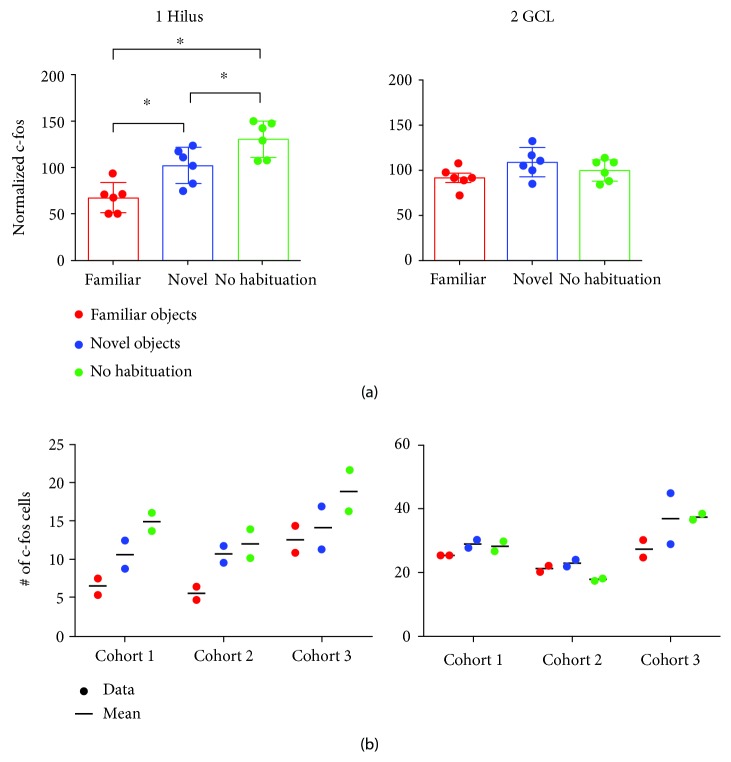
Hilar c-fos labeling is greatest in animals exposed to novelty and relatively unaffected in the GCL. (a) Hilar c-fos labeled cells are shown for the following 3 groups (mean, SD): mice exposed to familiar objects (FO), mice exposed to novel objects (NO), and mice with no prior exposure to objects (no habituation; NH). (a, 1) Hilar c-fos-labeled cell counts were normalized to the mean of the cohort. A one-way ANOVA showed a significant effect of exposure (*F*(2, 16) = 17.45; *p* < 0.0001) with the c-fos+ cells of the familiar group significantly less than the novel object group (*p* = 0.014) and no habituation group (*p* = 0.0001). The novel object group showed significantly fewer c-fos cells than the group with no habituation (*p* = 0.044). (a, 2) Normalized GCL c-fos+ cell counts did not exhibit statistical differences (one-way ANOVA, *F*(2, 16) = 2.33; *p* = 0.131). (b) Comparison of FO, NO, and NH for 3 cohorts. (b, 1) Hilar c-fos cell numbers are shown for all animals. The data are organized into 3 cohorts, with 2 animals/behavior for each cohort. Data for individual animals are designated by circles; means are indicated by bars between the circles. Red: FO; blue: NO; green: NH. Note the same pattern for each cohort, i.e., FO<NO<NH. (b, 2) c-Fos cell numbers are shown for the GCL. Data for each cohort do not indicate a consistent difference between FO, NO, and NH.

Analysis of hilar c-fos+ cells revealed that on average, sections from mice exposed to familiar objects had the lowest hilar c-fos protein expression, sections from the novel object group had more c-fos, and sections from the no habituation group had the highest hilar c-fos+ cell numbers (Figures 6 and 7(a)). In contrast, GCs showed little difference in c-fos protein expression in the 3 groups (Figures 7(a), 7(b)).

Note that the data shown in Figure 7(a) were pooled from 3 cohorts of animals. Each cohort is shown in Figure 7(b) to demonstrate that each cohort of animals showed the same pattern: the least hilar c-fos protein in the familiar objects group, more when exposed to novel objects, and the most when the mouse had never been exposed to objects before (Figure 7(b)). In contrast, each cohort did not show much difference in GCL c-fos protein expression when the familiar object, novel object, and no habituation groups were compared (Figure 7(b)).

### 3.3. Septotemporal Differences in c-Fos Protein in Response to Novel and Familiar Objects

**Figure 8:**
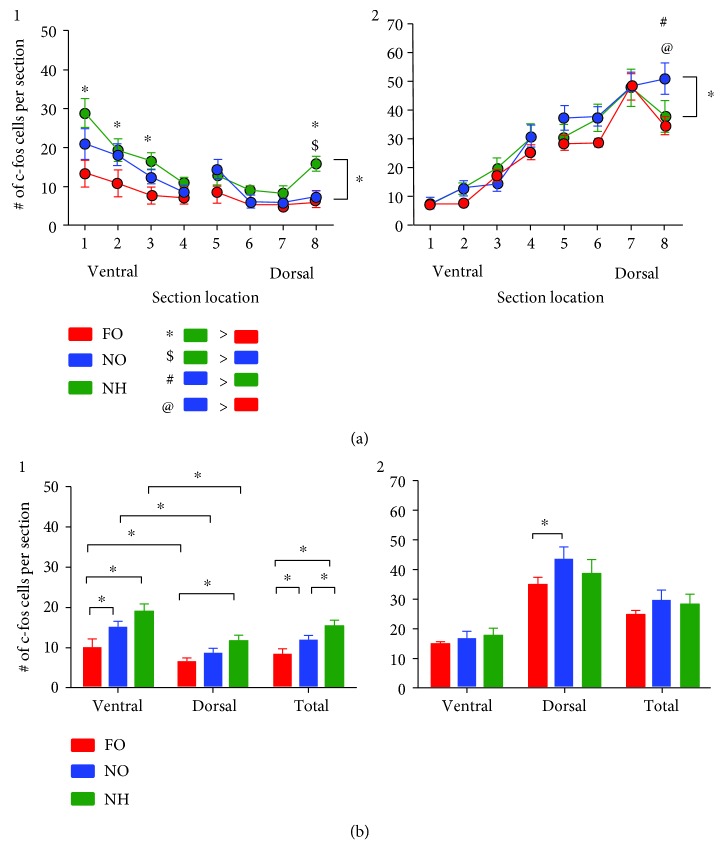
Septotemporal distribution of hilar and GCL c-fos expression in response to familiar objects (FO), novel objects (NO), and no habituation (NH). (a, 1) The numbers of c-fos+ hilar cells are shown for the same animals as Figure 5, plotted from the most ventral [34] to dorsal [56] levels. A two-way RMANOVA showed a significant effect of the task (FO: red; NO: blue; NH: green; *F*(2, 105) = 6.25; *p* = 0.011) and ventral-dorsal location (*F*(7, 105) = 19.06; *p* < 0.0001), and there was no interaction of factors (*F*(14, 105) = 1.68; *p* = 0.071). Tukey's post hoc tests showed significance (*p* < 0.05) at discrete locations along the septotemporal axis as indicated by symbols. Asterisk (∗) = NH > FO; dollar sign ($) = NH > NO; number symbol (#) = NO > NH; ^“^at^”^ sign (@) = NO > FO. (a, 2) Data are shown for GCL c-fos+ cells. A two-way RMANOVA showed no statistical effect of the task (*F*(2, 105) = 0.86; *p* = 0.441), but it showed a significant effect of the ventral-dorsal location (*F*(7, 105) = 105.50; *p* < 0.0001) with the most dorsal level exhibiting differences by Tukey's post hoc tests (*p* < 0.05). There was a significant interaction (*F*(14, 105) = 2.17; *p* = 0.014) because only the most dorsal level showed significant differences between the tasks by Tukey's post hoc tests. (b, 1) An analysis of the data from (a) is shown, pooling all ventral sections [5, 34, 35, 89] and comparing them to all dorsal sections [1, 57, 65, 73]. Totals are also shown (all 8 sections). A two-way ANOVA showed significant ventral-dorsal differences (*F*(1, 15) = 62.58; *p* < 0.0005) and a significant effect of the task (FO, NO, or NH: *F*(2, 15) = 29.52; *p* < 0.0001). There was no interaction of factors (*F*(2, 15) = 1.57; *p* = 0.253). Tukey's post hoc tests showed the significant differences of each task when comparisons were made of ventral vs. dorsal levels (e.g., ventral FO vs. dorsal FO, ventral NO vs. dorsal NO). For the ventral data, there were additional significant differences between FO and NO as well as FO and NH. For the dorsal data, FO vs. NH was significant by Tukey's post hoc test. A one-way ANOVA for pooled data (total) showed significant differences between tasks (*F*(2, 15) = 6.25; *p* = 0.011) and Tukey's post hoc tests were significant for all comparisons (FO vs. NO, FO vs. NH, and NO vs. NH). (b, 2) The ventral and dorsal data for the GCL are shown. A two-way ANOVA showed ventral-dorsal differences (*F*(1, 15) = 144.8; *p* < 0.0001) but no differences between tasks (*F*(2, 15) = 2.51; *p* = 0.126) and no interaction (*F*(2, 15) = 3.08; *p* = 0.091). Within ventral levels, there were no differences between tasks. For dorsal levels, FO vs. NO was significant. When all levels were pooled (total), a one-way ANOVA showed no significant differences between tasks (*F*(2, 15) = 1.75; *p* = 0.208).

To compare sections from all 3 groups (familiar objects, novel objects, no habituation) across the dorsoventral axis, sections were compared at 8 points along the septotemporal axis as in Figure 4. A two-way RMANOVA showed a significant effect of the dorsoventral axis, with ventral hilar c-fos higher than dorsal c-fos (Figure 8(a) 1). This is consistent with previous data showing that ventral hilar c-fos is higher than dorsal c-fos when an animal experiences a change in the environment [44, 48]. Tukey's post hoc tests showed that the group with no habituation had elevated c-fos cell numbers relative to the familiar object group in the 3 most ventral sections and also in the most dorsal section (Figure 8(a) 1).

In contrast to hilar c-fos, the differences in GCL c-fos along the dorsoventral axis showed more c-fos cell numbers in dorsal levels (Figure 8(a) 2). Thus, a two-way RMANOVA failed to show a significant effect of the behavioral task (familiar, novel, or no habituation) but there was a significant dorsal-ventral difference and an interaction of factors. The most dorsal level showed significantly more GCL c-fos protein in the novel object group relative to either of the other conditions (Figure 8(a) 2).

When data from all ventral sections were pooled in one group and all dorsal sections were pooled in a second group, ventral-dorsal differences were present for both hilus and GCL (Figure 8(b)). Tukey's post hoc tests showed significant differences for all ventral hilar cells when a specific task was compared with the same task in dorsal hilar cells (Figure 8(b) 1). On the other hand, GCL c-fos+ cells were not distinct when analogous comparisons were made (Figure 8(b) 2). Together, the data in Figures 8(a) and 8(b) suggest a greater effect of novelty on the hilus and MCs compared to the GCs. In addition, the inverse correlation of hilar c-fos and GCL c-fos (ventral hilar c-fos high; dorsal GCL c-fos high) agrees with prior studies where animals experienced a novel environment [44]. It was suggested [44] that this high degree of ventral hilar c-fos with dorsal GC c-fos could be related to the projection of ventral MCs to dorsal GCs [28, 61] which is discussed further below.

## 4. Discussion

In this study, we examined the influence of different types of novelty on c-fos protein in MCs and GCs of the adult mouse. The results advance our understanding of the role of MCs in the DG in several ways.

### 4.1. MCs and Different Types of Novelty

The 2 experimental designs allowed us to investigate the role of multiple types of novelty in c-fos protein expression. The first set of experiments demonstrated that hilar neurons, including MCs primarily, are activated after exposure to novel objects in the home cage. We also showed that novel objects only weakly activate GCs. However, the first experimental design did not allow us to distinguish whether the increased c-fos protein expression was due to the novelty of objects, because familiar objects were not assessed. One possibility was that the novelty of the objects did not lead to MC activation, but a change in wthe environment was the reason. In support of that possibility, a prior study in the rat showed that merely a change in environment could lead to c-fos protein expression in MCs [44]. Our first experiment also did not examine whether exposure to a new object is different from exposure to any object for the first time; in other words, is there an effect of novelty of the experience in addition to novelty of the object?

The second part of the study was therefore designed so that we could directly compare c-fos protein expression in response to novel objects, familiar objects, and a first exposure to objects. From this set of experiments, the results showed that novel objects increased hilar c-fos more than familiar objects, and that animals that had never been exposed to objects in their cage before showed the most hilar c-fos protein expression. This series of findings has important implications for understanding the potential role of MCs and other hilar cells in the detection of multiple types of novelty. First, these data implicate MCs in the circuitry that leads to discrimination of novel objects from familiar objects. In addition, it suggests that MCs are also part of the circuitry required for sensitivity to new experiences. Interestingly, the 2 kinds of novelty together (the novel experience and the novel object) appear to be somewhat additive. Thus, exposure to familiar objects leads to the activation of some MCs, but more MCs become activated after exposure to novel objects compared to familiar ones, and exposure to objects for the first time leads to the activation of the most MCs. Together, these results suggest that MCs are part of the circuitry used to detect multiple types of novelty, and that MC c-fos protein appears to increase with the degree of novelty.

The increase of hilar c-fos protein after novel object exposure occurred in all areas of the septotemporal axis of the DG, which is somewhat surprising given that the dorsal and ventral DG are thought to perform different functions, the dorsal more “cognitive” and the ventral more “limbic” [62, 63]. However, ventral MCs had more c-fos protein immunoreactivity than dorsal MCs, which we had found previously for other experiments [44, 48]. In the previous studies, we noted that ventral MCs have a substantial projection to distant lamellae in the dorsal DG, so ventral MCs may contribute to the “dorsal” functions of the DG and vice versa [44, 48]. These considerations make it unclear how well one can divide dorsal DG from ventral DG functionally, which has also been discussed for other reasons [64].

The anatomical connections of MCs suggest that they are well positioned to function in a circuit leading to novelty detection [26, 27]. Specifically, MCs receive concurrent sensory information from the EC via the PP as well as neuromodulatory inputs from the ascending brainstem-activating pathways including noradrenergic input from the locus coeruleus [65–67], serotoninergic input from the raphe nuclei [68, 69], cholinergic projections from the septum [70, 71], and additional extrinsic afferents [26, 72]. A subset of MCs has a low action potential threshold in response to PP stimulation, a characteristic particularly prominent in MCs with a dendrite in the molecular layer [27, 30]. Importantly, a recent study suggests that there is a direct projection to MCs from the EC [73], potentially innervating MC molecular layer dendrites. Notably, the primary area of the EC that innervated MCs was the lateral EC. This is interesting because the lateral EC encodes input related to new sensations [26] and therefore would be highly relevant to the detection of novelty. Thus, a direct pathway from the lateral EC to MCs could activate the MCs in response to novel input from sensory areas.

### 4.2. Rat vs. Mouse MCs

The results shed light on past findings in the adult Sprague-Dawley rat by extending them to C57BL/6 mice. Thus, in both species, ventral MCs appear to be highly sensitive to novelty. We previously showed in rats that removal from the animal facility and immediate perfusion in the laboratory induced MC c-fos ir, especially in ventral MCs [44]. Acclimating the rat to the laboratory decreased MC c-fos ir, but a significant fraction of MCs were still c-fos+ without acclimation. Rats were not placed in a new cage and were housed with their cage mate, like the mouse experiments. The numbers of c-fos+ MCs in rats were generally lower than the c-fos+ MCs in mice in the present study, when one compares the rat data to the control mice in the present study where objects were not presented.

In mice, although ventral MCs were most active, there were more MCs labeled in intermediate and dorsal levels than in the rat. Notably, sections were processed and oriented the same way in all studies so these potential differences could not explain the greater labeling of the mouse sections. Finally, in both rats and mice, GCs did express c-fos but mostly in the dorsal DG.

Together, these studies suggest that MCs are likely to be spontaneously active under many situations, and novelty could promote this activity. That interpretation is supported by physiological studies showing that MCs have a high rate of spontaneous excitatory input that facilitates action potential firing in vitro [74, 75] and exhibit spontaneous calcium signals in vivo using GCaMP transgenic mice recorded in a head-fixed preparation in vivo [29]. In contrast, GCs are relatively quiescent, which is very different from the frequent, large-amplitude spontaneous EPSPs from glutamatergic inputs that characterize MCs (Figure 8 of [76]; Figure 4 of [77]). Relative to MCs, GCs have more hyperpolarized resting potentials [59], and low rates of firing in vivo [3, 78, 79]. Rats and mice seem to share these differences between GCs and MCs, at least Sprague-Dawley rats and C57BL/6J mice.

### 4.3. Granule Cells

The results suggest that increased hilar c-fos was not accompanied by much change in GCL c-fos, which seems surprising if ventral MCs are active and project to dorsal GCs [28, 80]. However, GCs may require strong excitation to express c-fos, and MCs do not appear to excite GCs strongly under normal conditions. One reason is that the terminals from MCs are inhibited from releasing glutamate by cannabinoid (CB) type 1 receptors [81]. Also, GCs are ordinarily very hyperpolarized relative to the action potential threshold [59, 82, 83]. At their hyperpolarized resting membrane potentials, the unitary EPSPs from monosynaptically-connected MCs are relatively weak [80]. It has been proposed that GCs use sparse firing patterns to encode information [84], and these might be insufficient to induce c-fos protein expression. Importantly, the elevated activity of even a small number of GCs, reflected by increased c-fos protein expression, may be sufficient to influence CA3 because GCs can produce large unitary EPSPs in their targets with strong frequency facilitation [85–87]. Thus, the even weak induction of GCL c-fos expression may lead to the activation of area CA3.

In contrast to the findings presented here that suggest a role for MCs in the circuitry used to encode object novelty, recent studies have focused on the role of MCs in the context of spatial novelty [29, 31, 32]. Spatial novelty was also a focus of other studies where optogenetic silencing of dorsal MCs had no effect on the performance in an NOR task, but it did impair performance on a NOL task (in which the second object is moved instead of replaced; see Methods; [88]). Our results would suggest that MCs are involved in object novelty, so it is surprising that an effect in a NOR task was not shown. One reason may be the differences in the way NOR and NOL were done in the 2 studies. Another explanation is based on the study by Bui et al. (2018). The authors silencing dorsal MCs rather than silencing all MCs. That is relevant because dorsal MCs may be involved in spatial object tasks while object recognition tasks involve ventral MCs. Another factor that was different between studies was that we used the home cage for behavioral tests and social housing.

### 4.4. Putative Hilar GABAergic Neurons

Using double labeling of c-fos+ cells with an antibody to GluR2/3 showed that most c-fos+ hilar cells were likely to be MCs. We interpret the glutamatergic hilar cells, defined by GluR2/3 expression, as MCs because the only other possible glutamatergic cell type in the hilus is ectopic GCs, and these only occur in small numbers in the normal C57BL/6 mouse [57]. We presume that the other cells in the hilus are GABAergic because that has been documented in the past studies of the hilus in the mouse and all other species studies to date [27, 60, 89–91]. However, it is possible that the GluR2/3 negative cells were MCs that were not detected by the antibody to GluR2/3.

Interestingly, previous studies of rats showed that close to 100% of all hilar c-fos ir cells were MCs when animals were removed from their home cage and when they were acclimated to the laboratory [44]. Another study in rats that looked at acute restraint also found that all hilar c-fos+ cells were MCs [48]. The results presented here differ, since we observed that some hilar c-fos+ cells did not express GluR2/3. These cells were a small subset, approximately 10-15% of c-fos+ hilar cells and were evident in controls and in animals exposed to novel objects. One explanation for this discrepancy is species differences in the hilus in rats and mice that make the antibody less able to detect MCs in the mouse than in the rat. Another explanation is that the mouse has lower GluR2/3 expression than the rat, and this makes the antibody miss some hilar MCs because the antibody does not detect them. Another possibility is that mouse hilar GABAergic neurons correspond to GluR2/3-negative hilar cells.

If the GluR2/3-negative hilar neurons were GABAergic, it is important because the data would suggest that these putative hilar GABAergic neurons may be involved in the circuitry used for object recognition. Together, the results underscore the importance of the hilus to DG functions, specifically those that require the detection of novel aspects of the environment.

## 5. Conclusions

This study shows that c-fos protein expression increases in MCs of the adult mouse DG in response to novelty and novel objects. We also found that the majority of hilar neurons that expressed c-fos were MCs. The increased c-fos expression was strongest in the ventral DG. In contrast to MCs, GC c-fos expression was strongest in the dorsal DG, but less sensitive to novelty and novel objects. Taken together with past findings, there is a strong body of evidence that MCs play a role in the DG response to novelty.

## Data Availability

The data used to support the findings of this study are available from the corresponding author upon request.

## References

[1] Bakker A., Kirwan C. B., Miller M., Stark C. E. L. (2008). Pattern separation in the human hippocampal CA3 and dentate gyrus. *Science*.

[2] Knierim J. J., Neunuebel J. P. (2016). Tracking the flow of hippocampal computation: pattern separation, pattern completion, and attractor dynamics. *Neurobiology of Learning and Memory*.

[3] Leutgeb J. K., Leutgeb S., Moser M. B., Moser E. I. (2007). Pattern separation in the dentate gyrus and CA3 of the hippocampus. *Science*.

[4] Myers C. E., Scharfman H. E. (2009). A role for hilar cells in pattern separation in the dentate gyrus: a computational approach. *Hippocampus*.

[5] Anacker C., Hen R. (2017). Adult hippocampal neurogenesis and cognitive flexibility — linking memory and mood. *Nature Reviews Neuroscience*.

[6] McEwen B. S. (2003). Mood disorders and allostatic load. *Biological Psychiatry*.

[7] Tannenholz L., Jimenez J. C., Kheirbek M. A. (2014). Local and regional heterogeneity underlying hippocampal modulation of cognition and mood. *Frontiers in Behavioral Neuroscience*.

[8] Webster M. J., Knable M. B., O'Grady J., Orthmann J., Weickert C. S. (2002). Regional specificity of brain glucocorticoid receptor mRNA alterations in subjects with schizophrenia and mood disorders. *Molecular Psychiatry*.

[9] Wosiski-Kuhn M., Stranahan A. M. (2013). From pattern separation to mood regulation: multiple roles for developmental signals in the adult dentate gyrus. *Frontiers in Cellular Neuroscience*.

[10] Straube T., Korz V., Frey J. U. (2003). Bidirectional modulation of long-term potentiation by novelty-exploration in rat dentate gyrus. *Neuroscience Letters*.

[11] Kitchigina V., Vankov A., Harley C., Sara S. J. (1997). Novelty-elicited, noradrenaline-dependent enhancement of excitability in the dentate gyrus. *European Journal of Neuroscience*.

[12] Denny C. A., Burghardt N. S., Schachter D. M., Hen R., Drew M. R. (2012). 4- to 6-week-old adult-born hippocampal neurons influence novelty-evoked exploration and contextual fear conditioning. *Hippocampus*.

[13] Pan Y. W., Storm D. R., Xia Z. (2013). Role of adult neurogenesis in hippocampus-dependent memory, contextual fear extinction and remote contextual memory: new insights from ERK5 MAP kinase. *Neurobiology of Learning and Memory*.

[14] Scharfman H. E., Bernstein H. L. (2015). Potential implications of a monosynaptic pathway from mossy cells to adult-born granule cells of the dentate gyrus. *Frontiers in Systems Neuroscience*.

[15] Seib D. R., Chahley E., Princz-Lebel O., Snyder J. S. (2018). Intact memory for local and distal cues in male and female rats that lack adult neurogenesis. *PLoS One*.

[16] Chancey J. H., Poulsen D. J., Wadiche J. I., Overstreet-Wadiche L. (2014). Hilar mossy cells provide the first glutamatergic synapses to adult-born dentate granule cells. *The Journal of Neuroscience*.

[17] Piatti V. C., Schinder A. F. (2018). Hippocampal mossy cells provide a fate switch for adult neural stem cells. *Neuron*.

[18] Yeh C. Y., Asrican B., Moss J. (2018). Mossy cells control adult neural stem cell quiescence and maintenance through a dynamic balance between direct and indirect pathways. *Neuron*.

[19] Hunsaker M. R., Rosenberg J. S., Kesner R. P. (2008). The role of the dentate gyrus, CA3a,b, and CA3c for detecting spatial and environmental novelty. *Hippocampus*.

[20] Lemaire V., Aurousseau C., Le Moal M., Abrous D. N. (1999). Behavioural trait of reactivity to novelty is related to hippocampal neurogenesis. *European Journal of Neuroscience*.

[21] Montag-Sallaz M., Welzl H., Kuhl D., Montag D., Schachner M. (1999). Novelty-induced increased expression of immediate-early genes c-*fos* and arg 3.1 in the mouse brain. *Journal of Neurobiology*.

[22] Sierra-Mercado D., Dieguez D., Barea-Rodriguez E. J. (2008). Brief novelty exposure facilitates dentate gyrus LTP in aged rats. *Hippocampus*.

[23] Guzowski J. F. (2002). Insights into immediate-early gene function in hippocampal memory consolidation using antisense oligonucleotide and fluorescent imaging approaches. *Hippocampus*.

[24] Satvat E., Schmidt B., Argraves M., Marrone D. F., Markus E. J. (2011). Changes in task demands alter the pattern of *zif268* expression in the dentate gyrus. *The Journal of Neuroscience*.

[25] Schmidt B., Marrone D. F., Markus E. J. (2012). Disambiguating the similar: the dentate gyrus and pattern separation. *Behavioural Brain Research*.

[26] Scharfman H. E. (2016). The enigmatic mossy cell of the dentate gyrus. *Nature Reviews Neuroscience*.

[27] Scharfman H. E., Myers C. E. (2013). Hilar mossy cells of the dentate gyrus: a historical perspective. *Front Neural Circuits*.

[28] Buckmaster P. S., Wenzel H. J., Kunkel D. D., Schwartzkroin P. A. (1996). Axon arbors and synaptic connections of hippocampal mossy cells in the rat in vivo. *The Journal of Comparative Neurology*.

[29] Danielson N. B., Turi G. F., Ladow M. (2017). In vivo imaging of dentate gyrus mossy cells in behaving mice. *Neuron*.

[30] Scharfman H. E. (1991). Dentate hilar cells with dendrites in the molecular layer have lower thresholds for synaptic activation by perforant path than granule cells. *The Journal of Neuroscience*.

[31] GoodSmith D., Chen X., Wang C. (2017). Spatial representations of granule cells and mossy cells of the dentate gyrus. *Neuron*.

[32] Senzai Y., Buzsaki G. (2017). Physiological properties and behavioral correlates of hippocampal granule cells and mossy cells. *Neuron*.

[33] Guzowski J. F., Timlin J. A., Roysam B., McNaughton B. L., Worley P. F., Barnes C. A. (2005). Mapping behaviorally relevant neural circuits with immediate-early gene expression. *Current Opinion in Neurobiology*.

[34] Aggleton J. P., Brown M. W. (2005). Contrasting hippocampal and perirhinal cortex function using immediate early gene imaging. *The Quarterly Journal of Experimental Psychology Section B*.

[35] Albasser M. M., Poirier G. L., Aggleton J. P. (2010). Qualitatively different modes of perirhinal–hippocampal engagement when rats explore novel vs. familiar objects as revealed by c-Fos imaging. *The European Journal of Neuroscience*.

[36] Jenkins T. A., Amin E., Pearce J. M., Brown M. W., Aggleton J. P. (2004). Novel spatial arrangements of familiar visual stimuli promote activity in the rat hippocampal formation but not the parahippocampal cortices: a c-*fos* expression study. *Neuroscience*.

[37] VanElzakker M., Fevurly R. D., Breindel T., Spencer R. L. (2008). Environmental novelty is associated with a selective increase in Fos expression in the output elements of the hippocampal formation and the perirhinal cortex. *Learning & Memory*.

[38] Vann S. D., Brown M. W., Erichsen J. T., Aggleton J. P. (2000). Fos imaging reveals differential patterns of hippocampal and parahippocampal subfield activation in rats in response to different spatial memory tests. *The Journal of Neuroscience*.

[39] Wan H., Aggleton J. P., Brown M. W. (1999). Different contributions of the hippocampus and perirhinal cortex to recognition memory. *The Journal of Neuroscience*.

[40] Wan H., Warburton E. C., Zhu X. O. (2004). Benzodiazepine impairment of perirhinal cortical plasticity and recognition memory. *European Journal of Neuroscience*.

[41] Zhu X. O., Brown M. W., McCabe B. J., Aggleton J. P. (1995). Effects of the novelty or familiarity of visual stimuli on the expression of the immediate early gene c-fos in rat brain. *Neuroscience*.

[42] Zhu X. O., McCabe B. J., Aggleton J. P., Brown M. W. (1996). Mapping visual recognition memory through expression of the immediate early gene c-fos. *NeuroReport*.

[43] Zhu X. O., McCabe B. J., Aggleton J. P., Brown M. W. (1997). Differential activation of the rat hippocampus and perirhinal cortex by novel visual stimuli and a novel environment. *Neuroscience Letters*.

[44] Duffy A. M., Schaner M. J., Chin J., Scharfman H. E. (2013). Expression of c-fos in hilar mossy cells of the dentate gyrus in vivo. *Hippocampus*.

[45] Fa M., Xia L., Anunu R. (2014). Stress modulation of hippocampal activity – spotlight on the dentate gyrus. *Neurobiology of Learning and Memory*.

[46] McEwen B. S., Nasca C., Gray J. D. (2016). Stress effects on neuronal structure: hippocampus, amygdala, and prefrontal cortex. *Neuropsychopharmacology*.

[47] Patel A., Bulloch K. (2003). Type II glucocorticoid receptor immunoreactivity in the mossy cells of the rat and the mouse hippocampus. *Hippocampus*.

[48] Moretto J. N., Duffy A. M., Scharfman H. E. (2017). Acute restraint stress decreases c-fos immunoreactivity in hilar mossy cells of the adult dentate gyrus. *Brain Structure and Function*.

[49] Squire L. R., Wixted J. T., Clark R. E. (2007). Recognition memory and the medial temporal lobe: a new perspective. *Nature Reviews Neuroscience*.

[50] Jessberger S., Clark R. E., Broadbent N. J. (2009). Dentate gyrus-specific knockdown of adult neurogenesis impairs spatial and object recognition memory in adult rats. *Learning & Memory*.

[51] Takeda A., Tamano H., Ogawa T. (2014). Intracellular Zn^2+^ signaling in the dentate gyrus is required for object recognition memory. *Hippocampus*.

[52] Vogel-Ciernia A., Wood M. A. (2014). Examining object location and object recognition memory in mice. *Current Protocols in Neuroscience*.

[53] Haettig J., Stefanko D. P., Multani M. L., Figueroa D. X., McQuown S. C., Wood M. A. (2011). HDAC inhibition modulates hippocampus-dependent long-term memory for object location in a CBP-dependent manner. *Learning & Memory*.

[54] Mumby D. G., Gaskin S., Glenn M. J., Schramek T. E., Lehmann H. (2002). Hippocampal damage and exploratory preferences in rats: memory for objects, places, and contexts. *Learning & Memory*.

[55] Hsu S. M., Raine L., Fanger H. (1981). Use of avidin-biotin-peroxidase complex (ABC) in immunoperoxidase techniques: a comparison between ABC and unlabeled antibody (PAP) procedures. *Journal of Histochemistry & Cytochemistry*.

[56] Scharfman H. E., Mercurio T. C., Goodman J. H., Wilson M. A., MacLusky N. J. (2003). Hippocampal excitability increases during the estrous cycle in the rat: a potential role for brain-derived neurotrophic factor. *The Journal of Neuroscience*.

[57] Bermudez-Hernandez K., Lu Y. L., Moretto J. (2017). Hilar granule cells of the mouse dentate gyrus: effects of age, septotemporal location, strain, and selective deletion of the proapoptotic gene *BAX*. *Brain Structure and Function*.

[58] Leranth C., Szeidemann Z., Hsu M., Buzsaki G. (1996). AMPA receptors in the rat and primate hippocampus: a possible absence of GLUR2/3 subunits in most interneurons. *Neuroscience*.

[59] Scharfman H. E. (1992). Differentiation of rat dentate neurons by morphology and electrophysiology in hippocampal slices: granule cells, spiny hilar cells and aspiny ‘fast-spiking’ cells. *Epilepsy Research Supplement*.

[60] Scharfman H. E. (1999). The role of nonprincipal cells in dentate gyrus excitability and its relevance to animal models of epilepsy and temporal lobe epilepsy. *Advances in Neurology*.

[61] Ribak C. E., Seress L., Amaral D. G. (1985). The development, ultrastructure and synaptic connections of the mossy cells of the dentate gyrus. *Journal of Neurocytology*.

[62] Kheirbek M. A., Drew L. J., Burghardt N. S. (2013). Differential control of learning and anxiety along the dorsoventral axis of the dentate gyrus. *Neuron*.

[63] Strange B. A., Witter M. P., Lein E. S., Moser E. I. (2014). Functional organization of the hippocampal longitudinal axis. *Nature Reviews Neuroscience*.

[64] Fanselow M. S., Dong H. W. (2010). Are the dorsal and ventral hippocampus functionally distinct structures?. *Neuron*.

[65] Bijak M., Misgeld U. (1995). Adrenergic modulation of hilar neuron activity and granule cell inhibition in the guinea-pig hippocampal slice. *Neuroscience*.

[66] Harley C. W. (2007). Norepinephrine and the dentate gyrus. *Progress in Brain Research*.

[67] Walling S. G., Brown R. A., Miyasaka N., Yoshihara Y., Harley C. W. (2012). Selective wheat germ agglutinin (WGA) uptake in the hippocampus from the locus coeruleus of dopamine-*β*-hydroxylase-WGA transgenic mice. *Frontiers in Behavioral Neuroscience*.

[68] Bijak M., Misgeld U. (1997). Effects of serotonin through serotonin_1A_ and serotonin_4_ receptors on inhibition in the guinea-pig dentate gyrus in vitro. *Neuroscience*.

[69] Ghadimi B. M., Jarolimek W., Misgeld U. (1994). Effects of serotonin on hilar neurons and granule cell inhibition in the guinea pig hippocampal slice. *Brain Research*.

[70] Brunner H., Misgeld U. (1994). Muscarinic amplification of fast excitation in hilar neurones and inhibition in granule cells in the guinea-pig hippocampus. *The Journal of Physiology*.

[71] Deller T., Katona I., Cozzari C., Frotscher M., Freund T. F. (1999). Cholinergic innervation of mossy cells in the rat fascia dentata. *Hippocampus*.

[72] Leranth C., Hajszan T. (2007). Extrinsic afferent systems to the dentate gyrus. *Progress in Brain Research*.

[73] Azevedo E. P., Pomeranz L., Cheng J. (2019). A role of Drd2 hippocampal neurons in context-dependent food intake. *Neuron*.

[74] Scharfman H. E. (1993). Characteristics of spontaneous and evoked EPSPs recorded from dentate spiny hilar cells in rat hippocampal slices. *Journal of Neurophysiology*.

[75] Scharfman H. E., Schwartzkroin P. A. (1988). Electrophysiology of morphologically identified mossy cells of the dentate hilus recorded in guinea pig hippocampal slices. *The Journal of Neuroscience*.

[76] Scharfman H. E. (1995). Electrophysiological diversity of pyramidal-shaped neurons at the granule cell layer/hilus border of the rat dentate gyrus recorded in vitro. *Hippocampus*.

[77] McCloskey D. P., Hintz T. M., Scharfman H. E. (2008). Modulation of vascular endothelial growth factor (VEGF) expression in motor neurons and its electrophysiological effects. *Brain Research Bulletin*.

[78] Jung M. W., McNaughton B. L. (1993). Spatial selectivity of unit activity in the hippocampal granular layer. *Hippocampus*.

[79] Neunuebel J. P., Knierim J. J. (2012). Spatial firing correlates of physiologically distinct cell types of the rat dentate gyrus. *The Journal of Neuroscience*.

[80] Scharfman H. E. (1995). Electrophysiological evidence that dentate hilar mossy cells are excitatory and innervate both granule cells and interneurons. *Journal of Neurophysiology*.

[81] Chiu C. Q., Castillo P. E. (2008). Input-specific plasticity at excitatory synapses mediated by endocannabinoids in the dentate gyrus. *Neuropharmacology*.

[82] Staley K. J., Otis T. S., Mody I. (1992). Membrane properties of dentate gyrus granule cells: comparison of sharp microelectrode and whole-cell recordings. *Journal of Neurophysiology*.

[83] Williamson A., Patrylo P. R. (2007). Physiological studies of human dentate granule cells. *Progress in Brain Research*.

[84] Kesner R. P. (2007). A behavioral analysis of dentate gyrus function. *Progress in Brain Research*.

[85] Henze D. A., Wittner L., Buzsaki G. (2002). Single granule cells reliably discharge targets in the hippocampal CA3 network in vivo. *Nature Neuroscience*.

[86] Scharfman H. E., Kunkel D. D., Schwartzkroin P. A. (1990). Synaptic connections of dentate granule cells and hilar neurons: results of paired intracellular recordings and intracellular horseradish peroxidase injections. *Neuroscience*.

[87] Weisskopf M. G., Nicoll R. A. (1995). Presynaptic changes during mossy fibre LTP revealed by NMDA receptor-mediated synaptic responses. *Nature*.

[88] Bui A. D., Nguyen T. M., Limouse C. (2018). Dentate gyrus mossy cells control spontaneous convulsive seizures and spatial memory. *Science*.

[89] Amaral D. G., Scharfman H. E., Lavenex P. (2007). The dentate gyrus: fundamental neuroanatomical organization (dentate gyrus for dummies). *Progress in Brain Research*.

[90] Glykys J., Peng Z., Chandra D., Homanics G. E., Houser C. R., Mody I. (2007). A new naturally occurring GABA_A_ receptor subunit partnership with high sensitivity to ethanol. *Nature Neuroscience*.

[91] Morgan R. J., Santhakumar V., Soltesz I. (2007). Modeling the dentate gyrus. *Progress in Brain Research*.

